# Prevalence and factors associated with anaemia among children aged 6 to 59 months in Namutumba district, Uganda: a cross- sectional study

**DOI:** 10.1186/s12887-017-0782-3

**Published:** 2017-01-18

**Authors:** Fiona Kuziga, Yeka Adoke, Rhoda K. Wanyenze

**Affiliations:** Makerere University School of Public Health, College of Health Sciences, Makerere University, P.O. Box 7062, Kampala, Uganda

**Keywords:** Anaemia, Children, Namutumba district, Uganda

## Abstract

**Background:**

Anaemia is one of the major causes of death among children under five years in Africa, with a prevalence of 64.6% among pre-school children. In 2014, we conducted a cross-sectional study in Namutumba district in East-central Uganda to determine the prevalence and factors associated with anaemia among children aged 6 to 59 months.

**Methods:**

We conducted a household survey in 376 randomly selected households. One child aged 6 to 59 months was randomly sampled from each selected household. A structured questionnaire administered to an adult caregiver was used to collect household data. Blood was collected by finger or heel prick to estimate the haemoglobin level using a portable haemocue analyser. Anthropometric data including age, weight and height was collected for each child. A modified poisson regression model was used to determine the correlates of anaemia, prevalence ratios and their 95% confidence intervals (CI).

**Results:**

The prevalence of anaemia was high (58.8%) and was highest among children aged 12 to 23 months (68.5%) and males (61.3%). About 27.7% children were stunted. Children aged 6–11 and 12–23 months were more likely to be anaemic (APR = 1.12; 95% CI: 1.05–1.19 and APR = 1.12; 95% CI: 1.00–1.24 respectively), Resident of Magada and Namutumba (urban areas) were less likely to be anaemic (APR = 0.89; 95% CI: 0.87–0.91and APR = 0.86; 95% CI: 0. 85–0.88 respectively). Children of caretakers of a big family size (seven or more children) and with any formal education were less likely to be anaemic (APR = 0.94; 95% CI: 0.89–0.99 and APR = 0.93; 95% CI: 0.87–0.99). Stunting (HAZ scores) was a predictor of anaemia (APR = 1.07; 95% CI: 1.02–1.12).

**Conclusion:**

Anaemia is highly prevalent among children and there is need to invest in measures to prevent anaemia, especially among children in the rural areas.

## Background

Anaemia is a global public health threat especially in the developing countries [1–4]. In Africa, the prevalence of anaemia among preschool children is estimated at 64.6% [4], however, community-based estimates of anaemia prevalence among children in settings where malaria is endemic range from 49 to 76% [5]. Childhood anaemia is a preventable condition, which has serious consequences including growth retardation, poor immune system and increased susceptibility to diseases [6], and death [7–9] and has severe socio-economic consequences for families and communities.

Despite several interventions like de-worming, malarial presumptive treatment, provision of fortified complimentary foods or enriched foods [9, 10] that have been put in place to increase the iron status of children less than five years, anaemia remains a challenge in Uganda. It is estimated that 50% of children under five years in Uganda have anaemia. The prevalence of anaemia among children in the East Central region of Uganda, the setting for this study is even higher at 67.5% [11].

Namutumba district has had frequent episodes of malnutrition over the past five years, an indication of potentially high micronutrient deficiencies and anaemia. Estimating the prevalence of anaemia and especially understanding its determinants is critical in designing effective anaemia interventions. This study assessed the prevalence, nutritional and non-infectious disease factors associated with anaemia among children aged 6 to 59 months in Namutumba district, in East-central Uganda.

## Methods

This was a quantitative cross-sectional household survey.

### Study settings

This study was conducted in Namutumba district, in the east-central region of Uganda, a district that has suffered frequent episodes of malnutrition [12]. The main occupation of people in Namutumba district, across all sub-counties, is largely farming and major crops include; rice, groundnuts, sorghum, millet and cassava.

### Sample size calculation

The sample size was estimated using Bennett’s cluster survey sampling formula, assuming prevalence of anaemia of 67.5% among children aged 6 to 59 months, a design effect of 1.12, and standard error of 0.0255 [11]. Seven respondents were selected per cluster. A total of 376 children were recruited for the study and their mothers/caregivers were interviewed.

### Sampling procedure

Multi-stage sampling procedure was used. Three (50%) of the six sub-counties in the district were randomly selected. In each sub-county two parishes were randomly selected by ballot method and all villages within the selected parishes were selected. Overall, 54 villages were selected out of 348 villages within the district. Within each village (cluster) seven households were selected, using systematic random sampling. Household lists (sample frame) were obtained from Village Health Teams (VHTs). The sampling interval was determined by dividing the total number of households in the village by seven. In case the selected household did not have an eligible child, the next household was selected. Within each household one child aged 6 to 59 months was selected. In households with more than one child, one was selected randomly by the ballot method from the list of household members. All children aged 6 to 59 months who had stayed for at least 6 months in Namutumba district by the time of the study were eligible for enrollment.

### Data collection methods

Data collection was conducted from April to May 2014. Trained research assistants conducted face-to-face interviews with caregivers of the children using structured questionnaires. A caregiver was defined as the person who looks after or was in charge of the children’s well-being in the household.

### Measures

Some questions were adapted from the Uganda Demographic and Health Survey (socio demographic characteristics including; number of children in the household, number of people living in the household, respondents’ relationship with the child, age of caregiver and age of the child), while others were developed based on the study objectives. The pretested questionnaire was used to collect data on the non-nutritional factors which included; socio demographic characteristics and nutritional related factors such as household dietary diversity, food consumption, age at cessation of breast feeding and child health history.

### Haemoglobin level determination

Haemoglobin (Hb) levels were measured using the HaemoCue method (HaemoCue Hb® 301, Sweden) [13]. Hb determination was done within the homes by a team of trained health personnel. Blood was obtained from children by a heel or finger prick. The first drop of blood was wiped off and the next drop was collected into a disposable microcuvette. Blood was drawn carefully to avoid introducing bubbles. The Hb levels were displayed on a digital register on the HaemoCue 301 and recorded to the nearest 0.1 g/dl. To ensure hygiene and safety of the procedure, each set of accessory (lancet, microcuvette, gloves, and alcohol swabs) was used once. To ensure accuracy of the HaemoCue, a controlled cuvette was used on a daily basis [13]. Anaemia was classified into severe, moderate and mild according to the WHO/United Nations University/UNICEF cut –offs [14].

### Nutrition status assessment

The weight of each child was measured using a Seca weighing scale, which was calibrated to zero. Caregivers were asked to remove the child’s clothes or dress them in light clothes. The child was then told to stand on the weighing scale and the weight of the child was recorded. In case the child was not able to stand the caregiver of the child was told to stand on the weighing scale, his or her measurement was taken first then both child and caregiver were weighed. To obtain the weight of the child, the weight of the caregiver was subtracted from the measurement of both child and the mother. The weight was recorded to the nearest 0.1 kg.

To measure the height, the height board was placed either horizontally or vertically on a flat ground surface. The child’s caregiver was requested to remove the child’s foot wear and headgear then the child was assisted to stand against the height board. Children who were less than 2 years or less than 87 cm were measured while lying down (recumbent). The research assistant held the child’s body (head and legs) in the appropriate position to ensure accuracy. The height/length readings were recorded to the nearest 0.1 cm. The nutrition status of the children was determined using weight, height and age of the children. The WHO nutrition indices were used to classify the nutritional status as underweight (Weight for Age Z score, < −2.0 standard deviations), stunting (Height for Age Z score or Length for Age, < −2.0 standard deviations), wasting (Weight for Height Z score, < −2.0 standard deviations, or normal [15].

### Study variables

The dependent variable was anaemia (Haemoglobin levels <11 g/l). Independent variables included; socio-demographic factors, (number of children and number of household members) and other factors included; history of child health, child nutritional status, dietary diversity and age at cessation of breastfeeding, Maternal factors (education, age, and occupation), ﻿area of residence and Food consumption.

### Data analysis

Data were analysed using STATA version 12.0. Anaemia was categorized using the WHO [14] classification into; severe anaemia (Hb <7.0 g/dl), moderate anaemia (Hb level 7.0–9.9 g/dl), mild anaemia (Hb level 10.0–10.9 g/dl), and no anaemia (Hb levels ≥11 g/dl). Univariable analysis was conducted to describe the background characteristics of the households. Continuous variables, which included haemoglobin levels and age were summarized using proportions, means and standard deviations.

At bivariable analysis, the association between independent variables and anaemia was examined. The outcome variable was categorized into two; anaemia (Hb levels < 11 g/dl) and no anaemia (Hb levels ≥ 11 g/dl). A modified Poisson regression model with robust error variance was used to estimate prevalence ratios (PR) as a measure of association for the relationship between independent variables (maternal education, maternal occupation, maternal age ﻿, child’s sex, child’s age, and feeding practices, area of residence and history of child morbidity) and anaemia as the primary outcome. PR was used as a measure of association because of the high prevalence of the outcome (>10%), thus providing a better estimate of risk than the odds ratio (OR) [16, 17]. Adequacy of food consumption was categorized as; acceptable (score > 35), borderline (score 21.5–≤ 35) and poor (score <21.5), based on the UNWFP food consumption score [18]. The Food Consumption Score (FCS) is a composite score based on dietary diversity, food frequency, and relative nutritional importance of different food groups. The respondent is asked about frequency of consumption over a recall period of the past 7 days.

Nutrition status data was analysed using ENA for SMART (Version 2011). The WHO cutoff points were used to categorize the nutrition status of children including weight for height (WHZ) scores to determine whether the child was wasted, weight for age (WAZ) scores to determine whether the child was underweight, and height for age (HAZ) scores to determine whether child was stunted. The WHZ, WAZ and HAZ scores were categorized into malnutrition or normal nutrition status. The categorized nutrition status data was then exported into STATA 12.0 for further analysis. Prevalence ratios and their 95% confidence intervals were used to investigate associations between malnutrition indices (underweight, wasting and stunting) with anaemia. Statistical significance was established when the *p* – value was less than 0.05.

At multivariable analysis, a modified Poisson regression model with robust error variance was used to estimate adjusted prevalence ratios and their 95% confidence interval [17]. Variables, with p < 0.20 at bivariable analysis or potential confounders were considered for the multivariable analysis. The final multivariable model was selected by adding variables to the model using the forward selection method Adjustment for correlation within sub-counties was done at multivariable analysis.

## Results

A total of 378 children aged 6 to 59 months were identified for the study from Magada, Namutumba and Bulange sub-counties. Two eligible individuals declined participation. A total of 376 children participated in the study: 49.5% (186) were males while 50.5% (190) were females (Table 1). The mean age of the children was 24.9 months with a standard deviation (SD) of +/−13.8. Most of the children were residing in Magada sub-county 52.7% (198), followed by Namutumba subcounty 25.8% (97) (Table 1).

**Table 1:** Socio demographic characteristics of the study population

Variable	Number (*n* = 376)	Percentage (%)
Child characteristics		
Sex		
Male	186	49.5
Female	190	50.5
Age (months)		
6–11	74	19.7
12–23	127	33.8
24–35	89	23.7
36–47	53	14.1
48–59	33	8.8
Nutrition status of children		
Wasting (WHZ < −2 SD)	20^a^	5.3
Under weight (WAZ < −2 SD)	48^a^	12.8
Stunting (HAZ < −2 SD)	104^a^	27.7
Respondents characteristics		
Respondent relationship with child		
Mother	353	93.9
Father	7	1.9
Other caregiver	16	4.2
Respondents’ age (years)		
14–24	125	33.2
25–44	233	62
> =45	18	4.8
Respondents’ education level		
No education	56	14.9
Primary	219	58.2
Post primary	101	26.9
Respondents’ number of children		
1–3	180	47.9
4–6	135	35.9
≥ 7	61	16.2
Respondents’ Occupation		
Food Production	241	64.1
Business	67	17.8
Salary Jobs	29	7.7
Trade	14	3.7
Others	25	6.7
Area of residence		
Bulange Sub-county	81	21.5
Magada Sub-county	198	52.7
Namutumba Sub-county	97	25.8

^a^Indicator don’t reflect the number of normal children (total less than 376)

### Nutrition status of the children aged 6 to 59 months

The prevalence of wasting (WHZ < −2 SD) was 5.3% (*n* = 20), higher among males (7.5%) than females (3.2%). The prevalence of underweight (WAZ < −2SD) was 12.8% (48) while the prevalence of stunting (HAZ < −2SD) was 27.7% (104).

### Prevalence of anaemia among children aged 6 to 59 months in Namutumba District

**Fig. 1:**
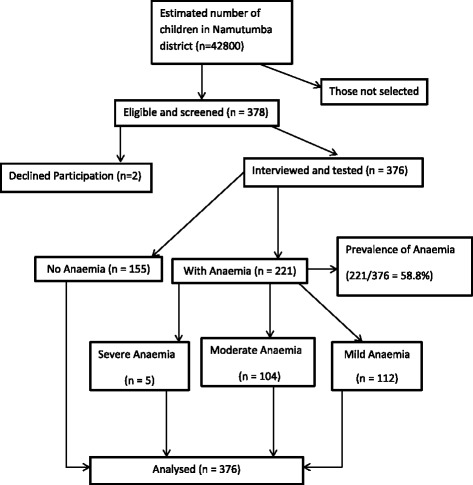
Recruitment Flow chart

Overall, 58.8% (221) of the children had anaemia (haemoglobin level below 11 g/dl). The haemoglobin levels ranged from 4.4 to 13.5 g/dl with mean haemoglobin level of 10.5 g/dl (SD 1.42). The proportion of children who had severe anaemia (Hb < 7 g.dl) was 1.3% (5), while those with moderate anaemia was 27.7% (104), and mild anaemia was 29.8% (112) (Fig. 1).

### Factors associated with child anaemia

At bivariable analysis, factors that were associated with anaemia included; child age, number of household members, number of children in a household, respondents education level, area of residence, child having suffered from fever in the past 3 months and stunting (Table 2).

**Table 2:** Association of factors with anaemia among children aged 6 to 59 months at bivariable analysis

Variable	Anaemia		Bivariable analysis	
	Yes (Y = 221) n (%age)	No (N = 155) n (%age)	Crude PR (95% CI)	*p*- value
Child age in months				
48–59	15(45.5)	18(54.5)	1.00	
36–47	23(43.4)	30(56.6)	0.99 (0.85–1.15)	0.852
24–35	49(55.1)	40(44.9)	1.07 (0.93–1.22)	0.352
12–23	87(68.5)	40(31.5)	1.16 (1.02–1.32)	0.023
6–11	47(63.5)	27(36.5)	1.12 (0.98–1.29)	0.089
Respondent’s education level				
No education	40(71.4)	16(28.6)	1.00	
Primary	127(58.0)	92(42.0)	0.92 (0.85–0.99)	0.047
Post primary	54(53.5)	47(46.5)	0.89 (0. 82–0.98)	0.021
Respondent’s age				
≥ 45	11 (61.1)	7 (38.9)	1.00	
25–44	131 (56.2)	102 (43.8)	0.92 (0.63–1.35)	0.672
14–24	79 (63.2)	46 (36.8)	1.03 (0.69–1.53)	0.867
Number of children				
1–3	118(65.6)	62(34.4)	1.00	
4–6	67(49.6)	68(50.4)	1.12 (1.04–1.21)	0.004
≥ 7	36(59.0)	25(41.0)	1.05 (0.95–1.16)	0.184
Number of household members				
1–5	109(64.5)	60(35.5)	1.00	
6–10	95(52.2)	87(47.8)	0.93 (0.87–0.99)	0.019
> 10	17(68.0)	8(32.0)	1.02 (0.91–1.15)	0.725
Area of residence				
Bulange Sub-county	64(79.0)	17(21.0)	1.00	
Magada Sub-county	107(54.0)	91(46.0)	0.86 (0.81–0.92)	<0.001
Namutumba Sub-county	50(51.6)	47(48.4)	0.85 (0.78–0.92)	<0.001
Height for Age				
Not Stunted	152(55.8)	120(44.1)	1.00	
Stunted	69(66.4)	35(33.6)	1.07 (0.99–1.14)	0.056
Food consumption score				
Acceptable	176(57.7)	129(42.3)	1.00	
Borderline	37(63.8)	21(36.2)	1.04 (0.96–1.13)	0.373
Poor	8(61.5)	5(38.5)	1.02 (0.87–1.21)	0.779
Fever past 3 months				
Yes	159(64.4)	88(35.6)	1.00	
No	62 (48.1)	67(51.9)	0.90 (0.841–0.965)	0.003

At multivariable analysis, factors that increased likelihood of anaemia included; number of children in a household, child’s age, sub county of residence, and stunting, while being educated was protective (Table 3).

**Table 3:** Overall factors associated with anaemia among children in Namutumba district

Variable	Anaemia		Bivariable analysis		Multivariable analysis^a^
	Yes (Y = 221) n (%age)	No (N = 155) n (%age)	Crude PR (95% CI)	*p*- value	Adjusted PR (95%CI)
Child age in months					
48–59	15(45.5)	18(54.5)	1.00		
36–47	23(43.4)	30(56.6)	0.99 (0.85–1.15)	0.852	0.99 (0.91–1.06)
24–35	49(55.1)	40(44.9)	1.07 (0.93–1.22)	0.352	0.92 (0.92–1.23)
12–23	87(68.5)	40(31.5)	1.16 (1.02–1.32)	0.023	1.12 (1.05–1.19)
6–11	47(63.5)	27(36.5)	1.12 (0.98–1.29)	0.089	1.12 (1.00–1.24)
respondent’s education level					
No education	40(71.4)	16(28.6)	1.00		
Primary	127(58.0)	92(42.0)	0.92 (0.85–0.99)	0.047	0.93 (0.87–0.99)
Post primary	54(53.5)	47(46.5)	0.89 (0. 82–0.98)	0.021	0.89 (0.78–1.01)
Number of children					
1–3	118(65.6)	62(34.4)	1.00		
4–6	67(49.6)	68(50.4)	1.12 (1.04–1.21)	0.004	0.91 (0.82–1.01)
≥ 7	36(59.0)	25(41.0)	1.05 (0.95–1.16)	0.184	0.94 (0.89–0.99)
Number of household members					
1–5	109(64.5)	60(35.5)	1.00		
6–10	95(52.2)	87(47.8)	0.93 (0.87–0.99)	0.019	0.99 (0.97–1.02)
> 10	17(68.0)	8(32.0)	1.02 (0.91–1.15)	0.725	1.06 (0.89–1.27)
Area of residence					
Bulange Sub-county	64(79.0)	17(21.0)	1.00		
Magada Sub-county	107(54.0)	91(46.0)	0.86 (0.81–0.92)	<0.001	0.89 (0.87–0.91)
Namutumba Sub-county	50(51.6)	47(48.4)	0.85 (0.78–0.92)	<0.001	0.86 (0.85–0.88)
Height for Age					
Not Stunted	152(55.8)	120(44.1)	1.00		
Stunted	69(66.4)	35(33.6)	1.07 (0.99–1.14)	0.056	1.07 (1.02–1.12)
Food consumption score					
Acceptable	176(57.7)	129(42.3)	1.00		
Borderline	37(63.8)	21(36.2)	1.04 (0.96–1.13)	0.373	1.04(0.89–1.20)
Poor	8(61.5)	5(38.5)	1.02 (0.87–1.21)	0.779	1.03 (0.95–1.13)
Fever past 3 months					
Yes	159(64.4)	88(35.6)	1.00		
No	62 (48.1)	67(51.9)	0.90 (0.841–0.965)	0.003	0.93 (0.85–1.01)

^a^Multivariable analysis results after adjusting for correlation within sub-counties

## Discussion

This study assessed the prevalence and non-infectious factors associated with anaemia among children aged 6 to 59 months in Namutumba district in Uganda. We found that 58.8% of the children aged 6 to 59 months were anaemic. Anaemia prevalence was highest among the male children (61.3%) and those aged 12 to 23 months (68.5%) followed by children aged 6 to 11 months, (63.5%). The prevalence of malnutrition was also very high with 27.7% of the children having chronic malnutrition/stunting and 5.3% with acute malnutrition/wasting. Factors associated with anaemia included; number of children in a household, respondents education level, area of residence, stunting, and child’s age.

The most prevalent conditions were moderate and mild anaemia, probably because mild and moderate anaemia is usually asymptomatic and may remain undetected and untreated [5, 19]. The prevalence of anaemia among the children was slightly lower than the East central regional average of 67.5% reported in the 2011 Uganda Demographic and Health Survey [11] but remains unacceptably high and is much higher than the prevalence in several sub-Saharan Africa countries such as Mali, Benin, Ethiopia, Senegal and the Middle East [9, 20–23]. Anaemia impairs the children’s immune system, leads to growth retardation and in severe cases may cause death if not well managed [24]. However, mothers can be taught and supported to prevent childhood anaemia at community level. The prevalence of anaemia in this region is of severe public health significance, well above the 40% WHO threshold, and clearly demands more aggressive interventions [14].

The high prevalence of anaemia in this district happens within the context of high levels of malnutrition with close to one third of the children having malnutrition. Stunting was significantly associated with anaemia unlike other nutrition indices such as wasting and underweight. Both stunting and anaemia (IDA) may be caused by malnutrition, and thus follow a similar causal pathway that is; feeding children less than four times a day and low dietary diversity [9, 25–28]. Both anaemia and stunting may result from failure to meet micronutrient requirements, including iron [29, 30]. Although the food consumption score was not significantly associated with anaemia, there was a high proportion of children with borderline and poor food consumption score with anaemia than those who had an acceptable food consumption score. This may be attributed to lack of diversified meals [9]. Presence of fever in the past three weeks was not significantly associated with anaemia in this study. However, the high levels of anaemia and chronic malnutrition might have been caused by infection diseases such as Malaria. Infections and especially malaria are major causes of anaemia in Africa [31].

According to the recent Uganda malaria indicator survey [32], malaria prevalence among children under five years was highest in the East Central region (where Namutumba lies), where 36% of children tested positive for malaria. Thus interventions to address anaemia and chronic malnutrition should be delivered in an integrated manner in order to comprehensively address the anaemia and various underlying problems.

Child age (6 to 23 months) was significantly associated with anaemia. The prevalence of anaemia was highest among the youngest age groups and generally reduced with increase in the age of the children [26, 27, 33, 34]. Iron stores are generally depleted among children by 6 months of age while the blood volume doubles from 4 to 12 months after birth. Thus, the dietary sources of iron are very important to keep up with this rapid rate of red blood cell synthesis and anaemia may result if the dietary sources are inadequate [1, 2].

The prevalence of anaemia was higher in the rural Bulange sub-county (79.0%) compared to Namutumba and Magada sub-counties probably due to higher levels of illiteracy and poorer access to health services, including health education [22]. Indeed, the prevalence of anaemia dropped with increase in the mother’s or caregiver’s level of education, as reported in other studies [1, 20, 26, 35, 36]. Although not significantly associated with anaemia, one third of the mothers/caregivers in this district did not know how to prevent anaemia, an indicator of the need for more education.

Families with seven or more children were less likely to have anaemic children compared to those with more children and the prevalence of anaemia increased with increasing number of children in the family. High maternal parity has been associated with anaemia as a high number of children impacts on the ability to feed them appropriately [37]. Most mothers, who had one to three children, were young mothers and two out of every three children among these young mothers had anaemia. Young mothers generally have challenges with child care due to limited resources and experience with child care and their children may have poorer health outcomes [38].

These findings highlight anaemia and underlying malnutrition among children as major challenges among rural communities in Eastern Uganda. There is need to concomitantly prioritize interventions to prevent anaemia and malnutrition in these communities in order to realize the much needed reduction of morbidity and mortality among infants and children in Uganda and other countries in sub-Saharan Africa.

### Study limitations

Some potential causes of anaemia (e.g.,; infections and other diseases), and the type of anaemia were not assessed. Also, as a cross sectional study, causality could not be established for any of the associated variables.

### Strength of the study

This household survey highlights the magnitude of anaemia in a rural district, one of the major and persistent contributors to childhood morbidity and mortality in Uganda and sub-Saharan Africa that has not received adequate attention.

## Conclusion

There was a very high prevalence of child anaemia noted in this rural district of Uganda, most prevalent among children aged 12 to 23 months. The prevalence of chronic malnutrition was also high and was associated with anaemia. There is need to invest in age specific measures to prevent anaemia, including routine screening and management, especially among children 6 to 23 months, children in the rural areas and those with low caregiver education.

## Abbreviations

APR: Adjusted prevalence ratio
CI: Confidence interval
ENA: Emergency nutrition assessment
FCS: Food consumption score
HAZ: Height-for-age Z score
Hb: Haemoglobin
IPTc: Intermittent preventive treatment for children
IRB: Internal review board
ITN: Insecticide treated nets
PR: Prevalence ratio
SD: Standard deviation
SMART: Standardized monitoring assessment relief transitions
UN WFP: United Nations World Food Programme
UNICEF: United Nations Children’s Fund
VHTs: Village health teams
WAZ: Weight-for-age Z score
WHO: World Health Organisation
WHZ: Weight-for-Height Z score
